# A novel approach to regulate glucose uptake in an anaplastic thyroid cancer cell line

**DOI:** 10.1530/EC-24-0336

**Published:** 2025-01-02

**Authors:** Shabnam Heydarzadeh, Ali Asghar Moshtaghie, Maryam Daneshpour, Mehdi Hedayati

**Affiliations:** ^1^ Cellular and Molecular Endocrine Research Center, Research Institute for Endocrine Sciences, Shahid Beheshti University of Medical Sciences, Tehran, Iran; ^2^ Department of Biochemistry, Falavarjan Branch, Islamic Azad University, Isfahan, Iran

**Keywords:** curcumin, anaplastic thyroid cancer, glucose uptake, glucose transporters, necrosis, apoptosis

## Abstract

**Abstract:**

**Aims and background**

Curcumin’s function in affecting cancer metabolic reprogramming remains poorly understood. Herein, we aimed to elucidate a novel link between curcumin and the glucose uptake metabolism and the status of glucose transporters (GLUTs) in the SW1736 cell line derived from anaplastic thyroid cancer.

**Materials and methods**

The 3-(4,5-dimethylthiazol-2-yl)-2,5-diphenyltetrazolium bromide test and flow cytometry were employed to test cell viability and cell death. For glucose uptake detection, the ‘glucose oxidase/peroxidase 4-aminoantipyrine’ enzymatic colorimetric assay was applied to measure the direct glucose levels inside the cells. Determination of GLUT1 and GLUT3 mRNA and protein expression in SW1736 cells was performed by qRT-PCR and western blotting. In addition, the scratch wound healing assay was conducted for cell migration.

**Results**

The data indicated that curcumin-induced cell death is independent of apoptosis in this type of thyroid cancer cell line. Furthermore, significantly reduced GLUT1 and GLUT3 expression was observed after treatment with curcumin, resulting in the inhibition of glucose uptake (*P* < 0.05). Scratch assay indicated the inhibition of cell migration in SW1736 cells treated with curcumin (*P* < 0.05).

**Conclusion**

It can be concluded that GLUTs, as metabolic targets, can be blocked specifically by curcumin for thyroid cancer prevention. Curcumin, as a promising anti-cancer agent, inhibits the growth of SW1736 anaplastic thyroid cancer cells by regulating the glucose uptake pathway and cell death. Altogether, these results suggest that the glucose pathway may be an essential target for therapeutic intervention to sensitize tumor cells to the cell death process by inhibition of glucose transporters.

**Statement of translational relevance:**

As described in our previously published article, ‘Regulators of glucose uptake in thyroid cancer cell lines’, non-oncogene addiction is one of the recent principles of cancer therapy. Moreover, herein, for the first time, we decided to investigate the anti-thyroid cancer effects of a bioactive phytochemical and establish a signaling link between curcumin and the glucose uptake metabolism of anaplastic thyroid cancer cells. This title covers the entire range of the biological sciences, and we attempt to fill the gaps between cell biology, medicine, cancer and plant knowledge. The influence of cancer on human society is indescribable. Human studies are often contradictory around the effects of polyphenols against cancer. We believe that it is important to draw boundaries between the usages of polyphenols in cancer prevention versus cancer treatment. We anticipate that this study’s findings will contribute to a better understanding of the mechanism underlying curcumin’s anti-cancer properties, providing a theoretical foundation for the future clinical application of curcumin-related medications.

## Introduction

Glucose metabolism serves as an essential physiological process that directly influences signaling pathways involved in cell death and provides energy to support cell proliferation (1). Cancer cells’ metabolic reprogramming helps them grow and survive, which is significantly different from normal cells (2). Increased expression of glycolytic transporters and enzymes is associated with cancer cells’ hyperproliferative and chemoresistant behavior. To satisfy the need for glucose required for elevated glycolysis in highly aggressive cancer cells, a significant quantity of glucose transporter (GLUT)-1 is necessary to ensure efficient absorption (3, 4, 5). GLUT1 and GLUT3 are the primary glucose transporters implicated in the oncogenesis of thyroid cancer, and their expression is much higher in malignant tissues than in normal tissues. They hold great potential as focus points for the research and development of cancer preventative treatments (6, 7, 8, 9, 10, 11, 12).

Previous research has indicated that curcumin can effectively reduce blood glucose levels in rats (13) and improve blood glucose and insulin sensitivity in mice (14). Studies on skeletal muscle (15) and myotubes (16) suggest a connection between curcumin and glucose metabolism, as curcumin has been shown to stimulate glucose uptake. While curcumin has been demonstrated to lower blood glucose in animal models of diabetes, there are limited studies on its effects on glucose uptake in tissue and cell models (17). However, the impact of curcumin on cancer cell glucose metabolism and whether inhibiting this response affects its anticancer properties have not been explored. Another potential mechanism for curcumin’s confirmed anticancer effects is its direct influence on the GLUT1 transport activity (18, 19). Numerous tumors express excessive GLUT1 and have high glycolytic rates (20, 21). According to reports, curcumin directly and dose-dependently suppresses GLUT1 activity in various cell lines. Curcumin immediately reduces the *V*_max_ of 2-deoxyglucose (2DG) absorption; this effect is reversible and decreases over time. A potential mechanism for the known anticancer effects of curcumin is the inhibition of glucose transporters’ activity, which may weaken cancer cells that overexpress GLUTs (22). These findings imply that the glycolytic pathway may be a significant therapeutic target for enhancing tumor cell sensitivity to cell death by inhibiting glucose transporters (23).

**Figure 1:**
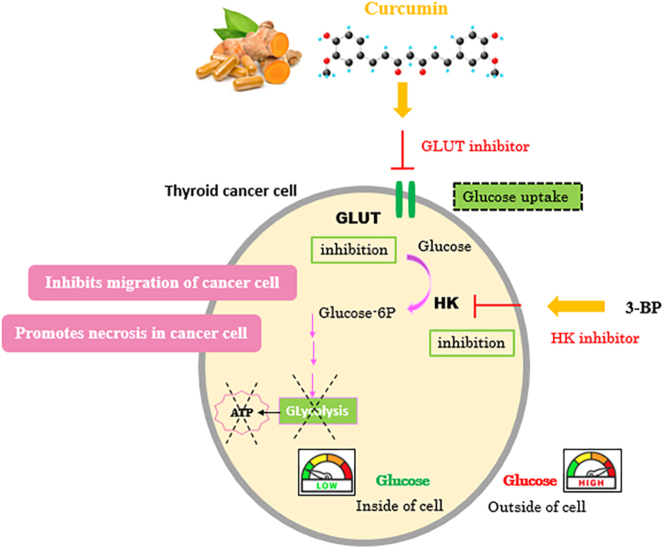
Effect of curcumin on glucose metabolism. Curcumin acts as a glucose transporter inhibitor, which can lead to the inhibition of glucose uptake, inhibition of cancer cell migration and promotion of necrosis in cancer cells.

Given the limited effectiveness of conventional approaches in predicting the prognosis of recurrent anaplastic thyroid cancer, it is imperative to gain a deeper understanding of the underlying molecular mechanisms and identify new agents to enhance therapeutic strategies. Herein, we aimed to investigate the effect of curcumin on thyroid cancer glucose metabolism and elucidate underlying mechanisms (Fig. 1). Clearly, we have a long way to go before we thoroughly comprehend how curcumin induces cell death. The present study investigated curcumin’s effect on glucose uptake (the Warburg effect) in a thyroid cancer cell line by assessing glucose uptake and GLUT1 and GLUT3 gene and protein expression levels. In this research, we further demonstrated the effect of curcumin on cell death. According to our knowledge, this is the first study to establish a link between curcumin and the glucose uptake metabolism of thyroid cancer. We propose that incorporating dietary phytochemicals in the early stages of cancer development may be deemed a promising approach for preventing cancer.

## Materials and methods

### Cell culture

The anaplastic thyroid cancer (ATC)-derived human thyroid cancer cell line utilized in this investigation was SW1736 (obtained from the Iranian Biological Resource Center in Tehran, Iran). This cell line exhibiting epithelial morphology has the accession cell no. IBRC C10311. Short tandem repeat analysis was used to confirm the authenticity of this cell line and test for the absence of mycoplasma. As previously mentioned, SW1736 cells were grown (24, 25). Cells were cultured in RPMI-1640 medium (BioIdea, Iran) with 10% FBS and penicillin (100 units/mL)/streptomycin (100 μg/mL) (Gibco, USA) and cultured at 37°C in a humidified incubator containing 5% CO_2_ (Memmert, Inc.). Experiments were conducted 48 h after seeding, and cell monolayers were routinely split twice a week.

### Drug preparation and treatment for glucose uptake assay

Stock solutions of 2.5 mM curcumin (purchased from Sigma-Aldrich, USA, CAS-No. 458-37-7, (HOC_6_H_3_(OCH_3_)CH=CHCO)_2_CH_2_) in DMSO (Merck, Germany) were prepared and refrigerated until use. Cells were treated with curcumin (purity (HPLC), % curcumin: ≥80% and total purity (HPLC), total curcuminoids: ≥94%) or without it. HPLC method details are as follows: column: LiChrospher® 100 RP-8 end-capped (5 μm) LiChroCART® 250-4. 150837, wavelength: 430 nm and mobile phase: citric acid and THF. The ultimate DMSO concentration in each experiment was less than 0.1% (v/v). For assessing cell cytotoxicity, 96-well plates were utilized, while 6-well plates were used for RT-PCR, western blot, apoptosis, migration and glucose uptake assays.

### Estimation of cell survival

The 3-(4,5-dimethylthiazol-2-yl)-2,5-diphenyltetrazolium bromide (MTT) assay, a widely used method for determining cell survival, was employed to assess the impact of curcumin on cancer cells, as outlined by Soni and coworkers (26). Cancer cells (1 × 10^4^ cells) were seeded in a 96-well tissue culture plate with varying concentrations of curcumin (0, 5, 10, 20, 50, 100, 150 and 200 μM) and incubated for 24, 48 and 72 h. MTT (Sigma-Aldrich, USA) was dissolved in PBS (Tamad Kala, Iran) at a concentration of 5 mg/mL, and 10% of the culture volume was added to each well in a sterile manner. The plate was then incubated at 37°C for 3 h to allow for the development of dark blue formazan crystals. The medium was carefully removed without disturbing the crystals, and 100 μL of MTT solvent were added to each well to dissolve the formazan crystals. The plates were then analyzed at a wavelength of 570 nm using a Multiskan EX microplate reader from Thermo Scientific (USA), and cell viabilities were compared to the control group. The IC_50_ values were calculated using a four-parameter logistic regression analysis in GraphPad Prism and confirmed using online calculators. All IC_50_ values were determined at the 48-h time point.

### Scratch wound healing assay

SW1736 cells were tested for cell invasion in an *in vitro* wound healing assay using curcumin or a vehicle control. Briefly, 8 × 10^5^ cells/well were seeded in 6-well plates overnight. With a pipette tip, a 1-mm-wide linear wound was made across the middle of each well. Following a PBS wash to get rid of the cell debris, wounded monolayers were subsequently cultured in RPMI-1640 containing curcumin. Three areas were randomly chosen for each well throughout the length of the wound to be photographed under phase-contrast microscopy. Following photography, the cells were incubated for 24 h at 37°C in a humidified incubator. Subsequently, the ImageJ software was employed to analyze the images.

### Detection of apoptosis

Changes in cell membrane permeability, an early indicator of apoptosis, can be detected with the annexin V-FITC apoptosis assay and propidium iodide (PI) staining. To summarize, apoptosis in cells was identified with the use of the annexin V-FITC Apoptosis Detection Kit (BioLegend, USA, Cat. no. 640906). For 48 h, cells were exposed to 50 μM curcumin. Subsequently, the cells were harvested using 0.25% trypsin–EDTA (Biosera, USA), followed by centrifugation at 1,400 rpm for 5 min. Both the attached and floating cells were collected, washed twice with ice-cold PBS and then resuspended in binding buffer (1×) at a concentration of 1 × 10^6^ cells/mL. From the solution containing 5 × 10^5^ cells, a portion was transferred to a culture tube. In order to stain the cells, annexin V-FITC and PI were added. Cells were vortexed and left to incubate in darkness for 15 min at RT (25°C). Finally, 1× binding buffer was added to each sample of cells in the tube. Flow cytometry was utilized to analyze apoptosis using the BD FACSCalibur device (BD Biosciences, USA).

### Glucose uptake assay

Here, we implemented a novel method for detecting glucose uptake in cultured cells, which involved the direct measurement of glucose levels. This method of glucose absorption detection has several advantages over current methods, including the absence of radioisotope materials, direct glucose uptake detection without the use of structurally modified glucose derivatives and detection with minimal culture condition changes and washing steps. To achieve this, we temporarily inhibited hexokinases (HKs) using the inhibitor 3-bromopyruvate (3-BP). The cells were treated with curcumin and incubated for 48 h in 6-well plates, after which the culture medium was removed. The HK 2 (HK-II) inhibitor 3-BP (200 μM) was added to exponentially growing cells for 30 min in a humidified incubator at 37°C. Following a 30-min incubation period, the cells were washed and resuspended in PBS to eliminate any residual glucose in the culture media before analysis. The cells were then lysed using a buffer containing 1 M Tris–base (pH = 7.6), MgCl_2_ (0.5 mM) and Triton X-100. Glucose uptake was detected by directly measuring the glucose levels inside the cells using the glucose oxidase (GOD)/peroxidase 4-aminoantipyrine (PAP) enzymatic photometric test, which involves enzymatic oxidation by glucose oxidase. The colorimetric indicator, quinoneimine, is produced from 4-aminoantipyrine and phenol by hydrogen peroxide under the catalytic action (Tinder’s reaction).

To determine glucose uptake, we optimized a method that utilized a culture medium containing glucose (RPMI-1640). In this method, the cells were treated with curcumin, separated and washed, and the glucose concentration in the culture media was subsequently measured. To validate the results, the cells were lysed immediately, and the amount of intracellular glucose (8 × 10^5^ cells) was measured in parallel with the extracellular glucose. The values were reported in terms of milligrams of glucose per milligram of protein (glucose mg/protein mg) after measuring the amount of total protein in the samples. The results were presented as the percentage of glucose uptake in comparison with the control group. The intracellular and extracellular glucose levels were measured using the GOD enzyme method and PAP oxidation product colorimetric method. The method exhibited a sensitivity of 10 μg/mL, and the intra-assay precision (coefficient of variation (CV) %) of the measurement was less than 2.5%.

### RNA extraction, cDNA synthesis and quantitative reverse transcriptase real-time PCR for GLUTs

Total RNA was extracted from both treated and untreated (control) cells using a BioFact Total RNA Prep Kit (column type, Cat. no. RP101-050, Korea) in accordance with the manufacturer’s guidelines. To enhance purity, the total RNA was treated with DNase I prior to cDNA synthesis. The first-strand cDNA was synthesized using the RevertAid H Minus First Strand cDNA Synthesis Kit (Thermo Scientific, USA). The qRT-PCR experiments were conducted using SYBR Green Master Mix (BioFact RT series, Korea). The primers for GLUT1, GLUT3, and β-actin genes were designed using the NCBI site and verified with the GeneRunner software (version 4.0) and NCBI Primer Blast (Table 1). The Rotor-Gene 6000 instrument (Corbett Research, Australia) was utilized, and the amplification conditions were as follows: 95°C for 10 min, 40 cycles at 95°C for 30 s, 61°C for 30 s and 72°C for 30 s. The data represent the mean ± standard deviation of triplicates within a single run. The relative expression of the GLUT1 and GLUT3 genes in each sample was determined using the 2^−ΔΔCT^ method based on the threshold cycle (*C*_t_), and CT was normalized by the β-actin reference gene.

**Table 1:** Primers used for qRT-PCR.

Target gene	NCBI access number	Primer sequence (5′–3′)	Tm	GC%	Product length (bp)
*GLUT1*	NM_006516.4	Forward: GTC​TGG​CAT​CAA​CGC​TGT​CTT​C	62.06	54.55	126
		Reverse: AAC​AGC​GAC​ACG​ACA​GTG​AAG	61.13	52.38	
*GLUT3*	NM_006931.3	Forward: ATG​CAC​ATA​GCT​ATC​AAG​TGT​G	56.58	40.91	102
		Reverse: GAC​CCT​GCC​TTA​CTG​CCA​ACC​TA	64.11	56.52	
*β-actin*	NM_001101.5	Forward: GAT​CAA​GAT​CAT​TGC​TCC​TCC​T	57.65	45.45	108
		Reverse: TAC​TCC​TGC​TTG​CTG​ATC​CA	58.43	50	

### Western blot analysis for GLUTs

The MTT assay data led to the decision of treating cells with 50 μM curcumin. Following a 48-h incubation period, both the control and treated groups were harvested using trypsin, washed with PBS, lysed using a buffer (RIPA) (70 mM Tris–HCl; pH = 7.4, 100 mM NaCl, 0.5% sodium deoxycholate, 0.1% SDS, 1.5 μM Pefabloc) and incubated for 1 h on ice. The cell lysates were then subjected to centrifugation at 7,000 rpm and 4°C for 10 min, and the supernatant was collected. The Lowry technique was used to measure the protein concentrations, and the absorbance was detected at 630 nm (ELISA Reader Biotek-reflex80). Total proteins and the PageRuler ladder (Thermo Scientific) were separated by SDS-PAGE (Mini-PROTEAN Tetra Vertical Electrophoresis Cell, Bio-Rad, Marnes-la-Coquette, France). Then, bands were transferred (100 V, 90 min) onto a PVDF transfer membrane (Sigma-Aldrich, USA, Cat. no. mspvdf04530301). The membranes were incubated overnight on a shaker in TBST (at 4°C) with primary antibodies directed against GLUT1 (Biorbyt, UK, Cat. no. orb77334), GLUT3 (Biorbyt, UK, Cat. no. orb649935) and GAPDH (GeneTex, USA, Cat. no. GTX100118) after being blocked with 5% skimmed milk (Sigma-Aldrich, Germany, Cat. no. STBG4695V) in TBST (0.05% Tween-20 (Sigma-Aldrich, Germany, Cat. no. 8-17072-1000)) for 2 h at room temperature. Following three washes with TBST, the membranes were incubated with the appropriate horseradish peroxidase-conjugated secondary antibody at room temperature for 2 h. The incubation solution contained TBST with 1% milk. The substrate for membrane development was a 3,3′-diaminobenzidine solution (Merck, Germany, Cat. no. D8001) and 0.3% hydrogen peroxide (Merck, Germany). The process was stopped by rinsing the blot with water, and the blot images were recorded. Finally, the membranes were incubated with an ECL (ParsTous, Iran, Cat. no. REF B111420) substrate solution and visualized with an autoradiography film.

## Statistical analysis

Since in the cell culture method, we are dealing with a uniform cell population from a specific clone under fully controlled conditions, the data distribution is assumed to be normal. On the other hand, in order to increase the precision of the interventions, each intervention should be considered as a three-fold repetition. Therefore, an independent *t*-test was used to compare the two means and one-way ANOVA was used to compare the means in several groups, and the results were considered to be statistically significant when values of *P* were less than 0.05. The software GraphPad Prism 9 was used to conduct the statistical analysis. The data represent the mean ± standard deviation of triplicates within a single run.

## Results

### Concentration- and time-dependent effect of curcumin on SW1736 cell viability

**Figure 2:**
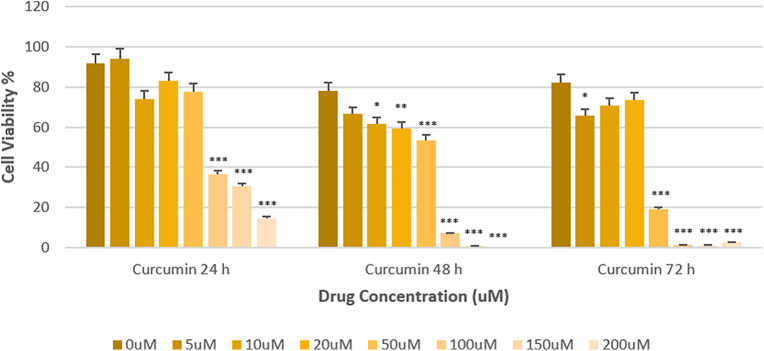
3-(4,5-Dimethylthiazol-2-yl)-2,5-diphenyltetrazolium bromide assay diagram for different concentrations of curcumin. Cells were treated in the absence (0 μM control) or presence of the indicated concentrations (5, 10, 20, 50, 100, 150 and 200 μM) of the curcumin for 24, 48 and 72 h. Statistical significance was determined using a one-way ANOVA followed by Tukey’s HSD post hoc test (*P < 0.05, **P < 0.01 and ***P < 0.001 for curcumin-treated groups versus the control group).

To investigate the effect of curcumin on cell viability, the MTT assay was utilized to determine the cell viability of ATC monolayer-growing cells. Fig. 2 demonstrates that curcumin exhibited strong inhibitory effects on SW1736 cells in a dose- and time-dependent manner, with an IC_50_ of 50 μM. As depicted in Fig. 2, curcumin significantly decreased cell viability after 48 h of exposure. Although a reduction in cell viability was observed at all experimental time points (24, 48 and 72 h), the reduction observed at 48 h was the most significant. Table 2 presents the mean cell viability, its standard deviation and the corresponding *P* value (Table 2).

**Table 2:** Mean cell viability, its standard deviation and the corresponding *P* value (* mean ± SD). The *P* values are reported in comparison with the control condition.

Concentration (μM)	24 h		*P* value	48 h		*P* value	72 h		*P* value
	Curcumin	Control		Curcumin	Control		Curcumin	Control	
5	*95.5 ± 4.5	92.8 ± 16.4	1.000	69.14 ± 7.03	84.6 ± 13.2	0.160	67.53 ± 5.78	85.6 ± 14.5	0.032
10	66.4 ± 16.4		0.099	64.64 ± 9.45		0.031	72.76 ± 6.50		0.244
20	81.3 ± 14.4		0.891	61.42 ± 9.49		0.008	72.79 ± 2.99		0.246
50	82.4 ± 15.3		0.932	52.21 ± 9.97		<0.001	20.12 ± 11.19		<0.001
100	40.0 ± 14.8		<0.001	8.13 ± 2.29		<0.001	1.21 ± 1.00		<0.001
150	29.4 ± 4.2		<0.001	1.33 ± 0.76		<0.001	1.391 ± 0.65		<0.001
200	12.9 ± 4.7		<0.001	0.38 ± 1.29		<0.001	2.48 ± 0.61		<0.001

### Curcumin decreased the glucose uptake in SW1736 anaplastic thyroid cancer cell line

**Figure 3:**
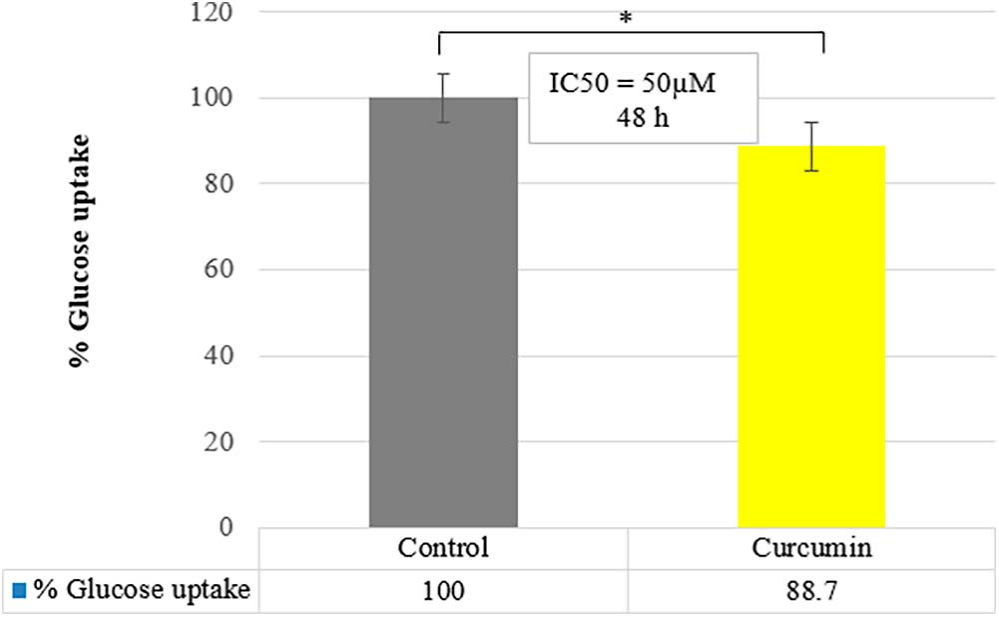
Glucose uptake assay in anaplastic thyroid cancer cells. Cells were treated with curcumin, and glucose uptake was measured using an enzymatic photometric test based on glucose oxidase oxidation. Statistical significance was determined using a *t*-test. *P* = 0.0235 indicates a statistically significant difference in glucose uptake between curcumin-treated cells and the control group (*P < 0.05).

**Figure 4:**
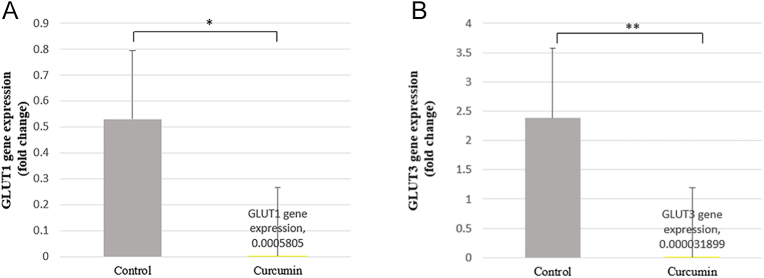
Effect of curcumin on glucose transporter (GLUT) 1 and GLUT3 expression in SW1736 cells. (A) GLUT1 and (B) GLUT3 mRNA expression levels were measured in SW1736 cells treated with curcumin compared to untreated controls. The data represent the mean ± standard error of triplicate measurements within a single experiment. Statistical significance was determined using a *t*-test (considered significant at *P* = 0.019 for GLUT1 expression and *P* = 0.001 for GLUT3 expression) (*P < 0.05; ** *P* < 0.01).

**Figure 5:**
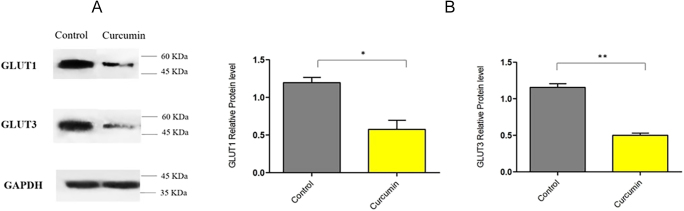
Effect of curcumin on GLUT1 and GLUT3 protein expression in SW1736 cells. (A) Representative western blot images showing GLUT1, GLUT3 and GAPDH protein levels in SW1736 cells treated with 50 μM of curcumin for 48 h. GAPDH was used as a loading control. (B) Relative protein levels of GLUT1 and GLUT3 normalized to GAPDH. Statistical significance was determined using a *t*-test. *P* = 0.0137 for GLUT1 and *P* = 0.0014 for GLUT3 indicate statistically significant differences compared to the control group. The original, full-length, and uncropped gel/blot images are included in the supplementary files (*P < 0.05; **P < 0.01).

The study of the glucose uptake process and its regulation, as well as the screening and characterization of medications that regulate glucose uptake during both normal and disease development, can all be accomplished with the help of this straightforward, sensitive and direct glucose uptake detection method (our patent in process). Fig. 3 shows the comparison of glucose uptake changes of the SW1736 cell line before and after treatment with curcumin in the concentration of 50 μM. According to the obtained results, glucose uptake was decreased significantly in the curcumin-treated group (*P* value = 0.0235). From Figs 4 and 5, it is clear that the curcumin-treated group has lower levels of both GLUT gene and protein expression compared to the control group. The low uptake of glucose was seen in the curcumin-treated group (Fig. 3), which had lower levels of GLUT 1 and GLUT 3 gene and protein expression (Figs 4 and 5). In addition, we have calculated the effect size (*d* ≈ 2.91) for the glucose uptake data between the curcumin and control groups. This indicates a very large effect size, suggesting that curcumin significantly affects glucose uptake compared to the control group (Table 3).

**Table 3:** Group differences and effect size.

Group	Mean (mg/mg pr)	SD	*P* value	% glucose uptake
Curcumin	65	3.5	0.0235	88.7
Control	73.3	2.0	1	100
Effect size (Cohen’s *d*)	–	–	–	2.91

### qRT-PCR and western blot analysis reveal curcumin-mediated GLUT1/GLUT3 inhibition

**Figure 6:**
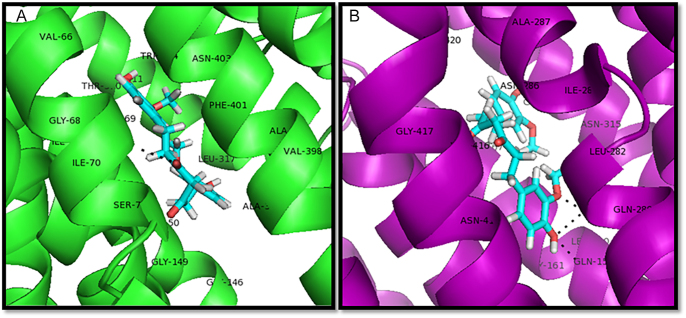
Curcumin docked into hGLUT1 and hGLUT3. PyMOL interaction for 4pyp (A) and 5C65 (B) receptors. The protein structures are homology models based on the 3D crystal structures of hGLUT1 and hGLUT3 and are shown as ribbons with side chains that contact curcumin, shown as sticks.

**Figure 7:**
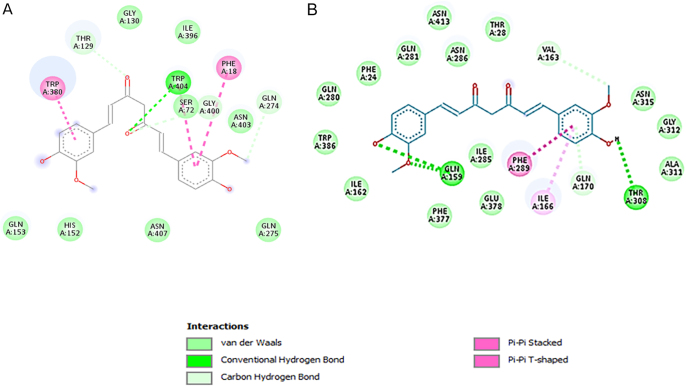
2D structure of different types of docking interactions between curcumin ligand and GLUT1 receptor (A) and GLUT3 (B) receptor (using Biovia Discovery Studio).

Curcumin significantly decreased both mRNA and protein expression of GLUT1 and GLUT3 in SW1736 cells after 48 h of treatment. QRT-PCR analysis revealed a substantial reduction in GLUT1 and GLUT3 mRNA levels (Fig. 4). GLUT1 and GLUT3 expression levels were downregulated in the sample group in comparison with the control group. These findings were further corroborated by western blotting, which demonstrated a marked decrease in GLUT1 and GLUT3 protein expression (Fig. 5). These results collectively indicate that curcumin effectively inhibits the expression of key glucose transporters in SW1736 cells, suggesting its potential to suppress glycolytic pathways. These findings provide evident data to verify the anti-glycolytic potential of our tested group with curcumin at the protein level. We have also included a figure illustrating the 3D and 2D structures of different types of docking interactions between the curcumin ligand and GLUT1 receptor and GLUT3 receptor (Figs 6 and 7).

### Curcumin promotes thyroid cancer cell death via inducing necrosis

**Figure 8:**
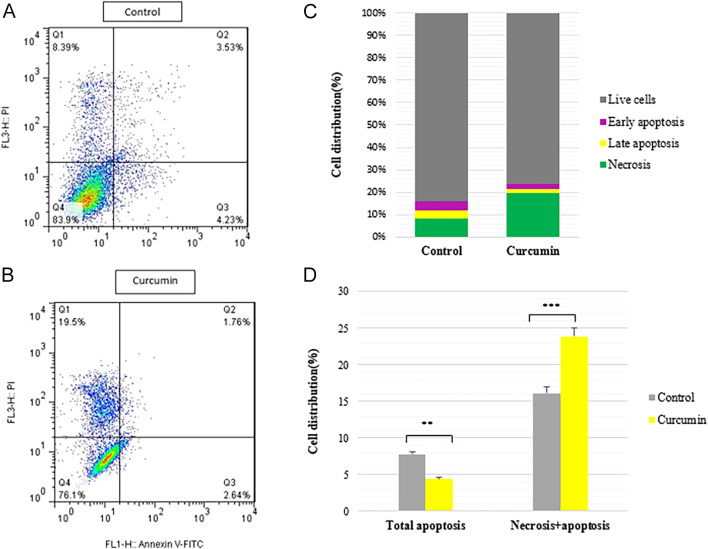
Flow cytometry analysis of curcumin-induced apoptotic cell death in SW1736 cells. Cells were treated with (50 μM curcumin for 48 h) or without curcumin. The apoptotic cells were determined by annexin V-FITC/PI analysis (A, B). Curcumin induces the necrosis of SW1736 cells. Percentage of living cells (lower left), necrosis cells (upper left), early apoptosis cells (lower right), and late apoptosis cells (upper right) are shown in the flow cytometry chart (C). Apoptotic and necrotic cell count determination by flow cytometry (D). Statistical significance was determined using an independent *t*-test. Results of cell distribution are represented as changes with respect to the control group (considered significant at *P* = 0.009 for total cell apoptosis and *P* < 0.001 for cells with necrosis + apoptosis) (** *P* < 0.01; *** *P* < 0.001).

The control of cancer cell death has important implications for cancer therapy; hence, research into curcumin’s effect on molecular targets has been intense. PI staining solution is a highly effective tool for identifying necrotic cells due to its selective interaction with nucleic acids. To investigate cell death, SW1736 cells were labeled with annexin V-FITC/PI and subsequently analyzed using flow cytometry. As depicted in Fig. 8, viable cells (annexin V−, PI−), necrotic cells (annexin V−, PI+), early apoptotic cells (annexin V+, PI−) and late apoptotic/necrotic cells (annexin V+, PI+) can be distinguished. Live cells (Q4) are negative for both annexin V and PI. At the earliest stage of apoptosis (Q3), cells bind annexin V but continue to exclude PI. They bind annexin V-FITC and stain strongly with PI during the late stage of apoptosis (Q2), and at the necrotic stage (Q1), cells bind annexin PI but continue to exclude annexin V. In necrotic cells, a hallmark feature is the loss of membrane integrity. This compromised membrane becomes permeable to molecules such as PI, which would not be able to enter a healthy cell with an intact membrane. Once PI enters the necrotic cell, it binds strongly to the DNA present in the nucleus.

As illustrated in Fig. 8, the curcumin-treated group exhibited a substantial increase in total cell necrosis (19.5%) compared to the control group (8.39%). According to the cell distribution graph, the percentages of live cells, apoptotic cells and necrotic cells compared between the curcumin-treated group and the control group are illustrated (Fig. 8). According to these findings, curcumin causes SW1736 cells to undergo cell necrosis. The antitumor effects induced by curcumin were associated with necrosis, with no significant induction of apoptosis. The data indicated that curcumin-induced cell death is independent of apoptosis in this type of thyroid cancer cell line.

### Curcumin inhibits cell migration in a concentration- and time-dependent manner

**Figure 9:**
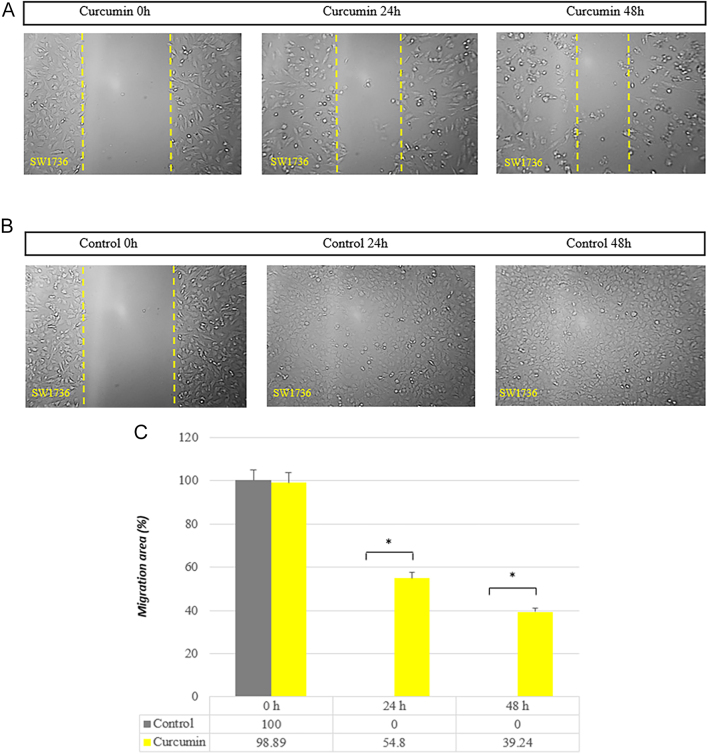
Inhibitory effect of curcumin on migration of SW1736 cell line in a monolayer wound healing model. Phase micrographs of cells treated (A) or not treated (B) with curcumin were taken at 0, 24 and 48 h after monolayer wounding (*P* = 0.037). Statistical significance was determined using an independent *t*-test. In the absence of curcumin (control groups 24 and 48 h), the cells migrate, resulting in a value of 0% for the available migration area (C). Data were quantitated as shown in the graph (*P < 0.05; ns, non-significant compared with control).

In order to gain insight into the cell migratory mechanism of curcumin, wound healing experiments were carried out. In the scratch experiment, as depicted in Fig. 9, the cells from the control group migrated to the scratch location right away. With more time and concentration than the control group, the treated group’s healing rate exhibited the reverse tendency (Fig. 9). In Fig. 9, the absence of black bars for controls at 24 and 48 h indicates that there is no measurable cell migration when curcumin is not present in the cell culture. The black bars represent the ‘available migration area’ in Fig. 9. This area essentially reflects the potential for cell movement or invasion within the assay. In the absence of curcumin (control groups at 24 and 48 h), the cells migrate, resulting in a value of 0% for the available migration area. This is why there are no black bars for these groups. There is an inverse relationship between these two terms. If there is high cell migration (many cells move through the scratch area), the available migration area (the potential space for movement) will be occupied by the migrating cells. This would translate to a smaller black bar in Fig. 9, signifying less remaining ‘available’ space. Conversely, if there is no cell migration (as in the curcumin treatment), the cells would not move through the assay. This would leave the entire ‘available migration area’ unoccupied, resulting in a full black bar in Fig. 9, representing the maximum potential space for migration.

## Discussion

Anaplastic thyroid cancer is a type of thyroid cancer that is rare but highly aggressive and is often unresponsive to traditional treatment methods (27). The ability of malignant cells to modify signaling and metabolic pathways to maintain well-known cancer markers is their remarkable feature. Unchecked growth depends on rewiring cellular metabolism to accommodate heightened energy and biosynthetic needs and coordinate several signaling pathways essential to cancer cell survival. Aerobic glycolysis, often known as the Warburg effect, is a fundamental adaptive metabolic response to the elevated biosynthetic demand of cancer cells. Even when enough oxygen is available, it is characterized by an enhanced absorption of glucose and a hyperproduction of lactate (28). Many cancer cells have significantly elevated glucose metabolism, and glycolysis takes over as the primary method for producing ATP in these cells (29, 30). Long recognized as the Warburg effect, this phenotype is frequently linked to the overexpression of glycolytic genes, such as GLUT1 and HKs (1). As a supportive anticancer treatment, clinical research is being done on the ability of calorie restriction or glucose restriction to reduce the increased glycolytic activity in cancer cells (31). Curcumin, as a natural compound, has gained attention for its potential anti-cancer properties. The objective of this study was to examine the effect of curcumin on glucose metabolism (the Warburg effect) in a thyroid cancer cell line and investigate the effect of curcumin on cell death. However, there is no specific research on the role of curcumin on glucose uptake and glucose transporters in anaplastic thyroid cancer cells. Further research is needed to investigate this topic.

Considering that several polyphenols, such as resveratrol and phloretin, have been reported to represent their anticancer effect dependent on the inhibition of glucose cellular uptake (32, 33, 34, 35), this study aimed to investigate the molecular effects of curcumin on glucose uptake and the gene and protein expression of glucose transporters in anaplastic thyroid cancer cell lines. Currently, there is limited research on the impact of curcumin on glucose uptake and glucose transporter expression in these cell lines. Our results indicated that curcumin can lead to a reduction in intracellular glucose. In addition, a recent study demonstrated the effect of curcumin treatment in combination with glucose restriction on intracellular alkalinization and tumor growth in hepatoma cells. The study found that curcumin treatment decreased glucose uptake in HepG2 cells (31). In a separate study, it was demonstrated that curcumin could inhibit glucose uptake and lactic acid levels in PTC cell lines B-CPAP, BHT-101 and KTC-1. Treatment with curcumin, with or without si-LINC00691 transfection, increased ATP levels and decreased glucose uptake, lactic acid levels and protein expression of LDHA and HK2 in B-CPAP cells. These findings suggest that curcumin may potentially inhibit the Warburg effect by regulating LINC00691 expression in B-CPAP cells (36). Taken together, our findings confirm the inhibition of glucose uptake through the downregulation of glucose transporters 1 and 3. In addition, we have demonstrated that the HK-II inhibitor 3-BP, which was used in the glucose uptake assay, may inhibit HK-II. These results suggest the potential use of this nutraceutical as a coadjuvant in the treatment of anaplastic thyroid cancer.

Previous research demonstrated that curcumin, a compound found in turmeric, has potent anti-thyroid cancer effects. In a study conducted by Zhang and coworkers, it was found that curcumin can trigger endoplasmic reticulum stress, leading to the death of B-CPAP cells, a type of papillary thyroid cancer cell line. In addition, the researchers were able to show that curcumin can induce apoptosis in B-CPAP cells of human papillary thyroid carcinoma. To achieve this, B-CPAP cells were treated with different concentrations of curcumin, and cell apoptosis was determined using annexin V/PI double staining (37). In a study carried out by Al-Mohanna and coworkers, it was found that the curcumin analog PAC can trigger apoptosis in CAL-62 anaplastic thyroid cancer cells through the mitochondrial pathway. The researchers observed that this process occurred in a time-dependent manner. According to the researchers, PAC was found to be effective in suppressing the epithelial-to-mesenchymal transition process in CAL-62 anaplastic thyroid cancer cells. This was achieved by upregulating the epithelial marker E-cadherin and reducing the levels of the mesenchymal markers N-cadherin, Snail and Twist1 (38).

Furthermore, Liang and coworkers conducted a study to investigate the impact of curcumin on the viability, migration and invasion of TPC 1 papillary thyroid cancer cells. Through modulation of the miR-301a-3p/STAT3 axis, their research demonstrated that curcumin could inhibit the viability, migration and invasion of TPC 1 cells (39). In a study conducted by Hong and coworkers, the researchers aimed to investigate whether curcumin could enhance docetaxel-induced apoptosis of ATC cells. The results showed that the combination of Curcumin and docetaxel led to a decrease in cell viability when compared to docetaxel or curcumin alone. Furthermore, curcumin treatment increased the docetaxel-induced apoptosis of ATC cells, as determined by annexin V staining and flow cytometry analysis (40). According to studies, polyethylene glycol has been found to trigger the anti-cancer effects of Curcumin nanoparticles in SW1736 thyroid cancer cells (41).

It is essential to emphasize that the mechanism of cell death can vary depending on the type of cell, compound’s concentration and treatment duration. Although apoptosis is a well-known mechanism by which curcumin induces cell death, it is not the only one. Necrosis, characterized by cell enlargement and rupture, can also result from curcumin treatment. The concentration of curcumin used in our investigation may have led to a different mechanism of cell death. In addition, the duration of the treatment may have played a role in the observed effect. It is also important to consider the specific characteristics of anaplastic thyroid cancer cells, as they may respond differently to curcumin compared to other cell types. In the case of curcumin treatment, it is possible that the compound may induce apoptosis initially, but then, the cells may undergo a switch to necrosis due to various factors. This could explain the difference in the mechanism of cell death observed in our investigation compared to previous studies.

Our findings are consistent with previous research conducted on obatoclax in anaplastic thyroid cancer cells. While there is limited recent research on the effect of curcumin on necrosis in anaplastic thyroid cancer cell lines, some studies have investigated the impact of curcumin on apoptosis in thyroid cancer cells, including papillary thyroid cancer cells. According to Champa and coworkers, obatoclax has been found to induce significant necrosis in anaplastic thyroid cancer cells. Unlike apoptosis, obatoclax is a compound that kills anaplastic thyroid cancer cells by inducing necrosis. The researchers demonstrated that obatoclax does not induce apoptosis but rather necrosis of thyroid cancer cells (42).

In a study conducted earlier, SW1736 and 8505C cells were treated with 50 μM DMSO, resveratrol, curcumin, genistein or EGCG for 48 h. The levels of cleaved PARP were then assessed as an apoptosis marker using western blotting. The results indicated a substantial reduction in ATC cell viability and an increase in the apoptosis process following treatment with phytochemicals in both analyzed cell lines. Using western blotting and an anti-cleaved-PARP antibody, apoptosis was the subject of investigation. Western blotting measures the expression of specific proteins involved in apoptosis, while flow cytometry measures the externalization of phosphatidylserine on the cell surface, an early event in apoptosis. Therefore, the results obtained from these two techniques may not always be consistent. Due to the mentioned reason, the dissimilarity seen in apoptosis results could be explained.

It is widely acknowledged that evaluating cell viability and apoptosis alone may not entirely represent the biological efficacy of a treatment. Therefore, in this study, we aimed to investigate the effect of curcumin on tumor aggressiveness by conducting a scratch wound healing assay. Our findings revealed, for the first time, that curcumin administration could induce a significant reduction in cell migration in SW1736 cells. This is an essential parameter as the few surviving cells could potentially lead to tumor regrowth and metastasis.

Our starting hypothesis was that curcumin represents its anti-cancer effect through inducing apoptosis pathways. These findings indicated that the effect of curcumin on the SW1736 cell line was independent of apoptosis, and the main effect was through inducing the necrosis pathway. There have been several instances in the literature where a correlation between ATP levels and the conversion of apoptosis to necrosis has been described. It is evident that ATP is required for the activation and function of caspases; therefore, energy is required to continue apoptosis. Necrosis can occur when ATP levels drop too drastically. Changes in the cellular energy charge may significantly influence the decision of the cell to die through apoptosis or necrosis (43). When ATP is depleted, decreased glucose availability, for example, may convert apoptosis to necrosis. To shift the death program toward either apoptosis or necrosis, controlled intracellular ATP depletion conditions were employed by preventing mitochondrial and/or glycolytic ATP synthesis, in combination with replenishing the cytosolic ATP pool with glucose. The results showed that when energy levels are rapidly depleted, cells that would typically undergo apoptosis are instead compelled to die by necrosis (44).

A study conducted on osteoblast cells found that treatment with curcumin resulted in a dose-dependent decrease in intracellular ATP levels. Furthermore, pre-treatment of cells with antimycin or 2DG to generate ATP depletion significantly altered the curcumin-induced apoptosis to necrosis in the 12.5−25 μM range. This concurs with earlier research that found ATP levels essential for the apoptosis/necrosis switching mechanism. According to the study, there may be a relationship between the dosage of curcumin and the rate of ROS formation, intracellular ATP levels and cell apoptosis or necrosis in osteoblast cells (45). It has been demonstrated that curcumin treatment can inhibit ATP synthase. However, due to low glucose uptake, the energy supply required for intracellular ATP synthesis is restricted (31).

Nonetheless, as a preliminary study, this research has limitations. We acknowledge the limitations of a single cell line and the importance of future studies that explore these effects in a wider range of cell lines and a wider range of curcumin dosages. We will investigate this mechanism further in additional thyroid cancer cell lines. In addition, we suggest that the effect of curcumin on other metabolic factors should be studied. It may also be worthwhile to investigate the effects of curcumin on other types of thyroid cancer, such as differentiated thyroid cancer. Finally, research could focus on optimizing the delivery of curcumin to cancer cells, as its poor bioavailability has been a limitation in previous studies. Due to the limited budget for evaluating ATP levels in this investigation, it has not been possible to perform it. However, it is in our mind to design and perform another investigation in our future projects with the aim of evaluating the effect of curcumin in combination with different types of other phytochemicals on ATP levels and compare this factor by glucose uptake and the apoptosis pathway in anaplastic thyroid cancer cells.

Future directions for research on curcumin and anaplastic thyroid cancer may include investigating the efficacy of curcumin in combination with other treatments, such as chemotherapy or radiation therapy, to enhance its anti-cancer effects. In addition, further studies could explore the mechanisms by which curcumin induces apoptosis in thyroid cancer cells, as well as its potential to inhibit metastasis and induce DNA damage. While this study suggests that curcumin may have potential as a therapeutic agent for ATC, further research is required to gain a comprehensive understanding of the mechanisms of action and efficacy of curcumin. Future studies should focus on optimizing the delivery of curcumin to cancer cells and investigating its effects in animal models and human clinical trials.

## Conclusion

In conclusion, curcumin treatment of SW1736 cancer cells reduced GLUT1 and GLUT3 expression levels, which led to a metabolic shift and reduced glucose uptake across the cell membrane. We concluded that the antitumor effects induced by curcumin were associated with necrosis and glucose uptake inhibition, with no significant induction of apoptosis. In this context, we propose a potential anti-invasive mechanism for curcumin in this investigation. By examining curcumin’s effect on anaplastic thyroid cancer cells, it was hypothesized that curcumin would exert an anti-invasion effect by reducing migration. We anticipate that the findings of this study will contribute to a better understanding of the mechanism underlying curcumin’s anti-cancer properties, providing a theoretical foundation for the future clinical application of curcumin-related medications.

## Declaration of interest

The authors declare that there is no conflict of interest that could be perceived as prejudicing the impartiality of the work reported.

## Funding

This work did not receive any specific grant from any funding agency in the public, commercial or not-for-profit sector.

## Author contribution statement

ShH and MH designed and drafted the manuscript, collected the references and carried out the primary literature search. AAM and MD modified the manuscript and participated in discussions. All authors read and approved the final manuscript.

## Data availability

Data will be made available on request.
